# Designed multi-stranded heme binding β-sheet peptides in membrane†
†Electronic supplementary information (ESI) available. See DOI: 10.1039/c5sc04108b


**DOI:** 10.1039/c5sc04108b

**Published:** 2015-12-17

**Authors:** Areetha D'Souza, Mukesh Mahajan, Surajit Bhattacharjya

**Affiliations:** a School of Biological Sciences , 60 Nanyang Drive , 637551 , Singapore . Email: surajit@ntu.edu.sg ; Fax: +65-6791-3856

## Abstract

Designed peptides demonstrating well-defined structures and functioning in membrane environment are of significant interest in developing novel proteins for membrane active biological processes including enzymes, electron transfer, ion channels and energy conversion. Heme proteins' ability to carry out multiple functions in nature has inspired the design of several helical heme binding peptides and proteins soluble in water and also recently in membrane. Naturally occurring β-sheet proteins are both water and membrane soluble, and are known to bind heme, however, designed heme binding β-sheet proteins are yet to be reported, plausibly because of the complex folding and difficulty in introducing heme binding sites in the β-sheet structures. Here, we describe the design, NMR structures and biochemical functional characterization of four stranded and six stranded membrane soluble β-sheet peptides that bind heme and di-heme, respectively. The designed peptides contain either ^D^P-G or ^D^P-^D^A residues for the nucleation of β-turns intended to stabilize multi-stranded β-sheet topologies and ligate heme with bis-His coordination between adjacent antiparallel β-strands. Furthermore, we have optimized a high affinity heme binding pocket, *K*_d_ ∼ nM range, in the adjacent β-strands by utilizing a series of four stranded β-sheet peptides employing β- and ω-amino acids. We find that there is a progressive increase in cofactor binding affinity in the designed peptides with the alkyl chain length of ω-amino acids. Notably, the six stranded β-sheet peptide binds two molecules of heme in a cooperative fashion. The designed peptides perform peroxidase activity with varying ability and efficiently carried out electron transfer with membrane associated protein cytochrome c. The current study demonstrates the designing of functional β-sheet proteins in a membrane environment and expands the repertoire of heme protein design.

## Abstract 2

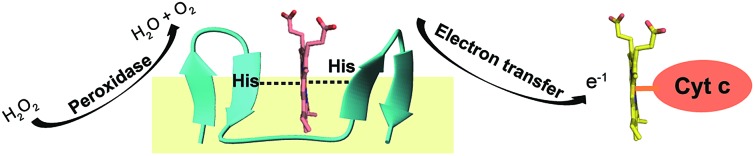
Structures and functions of designed multi-stranded heme binding β-sheet peptides carrying out peroxidase activity and electron transfer in membrane.

## Introduction

**Fig. 1:**
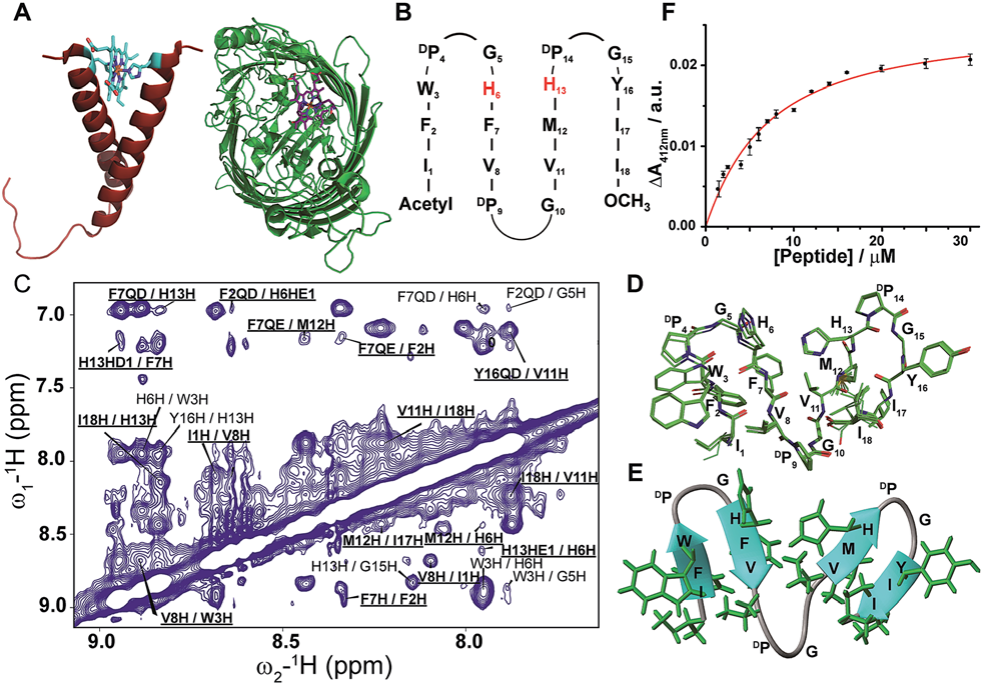
Design, structure and heme binding of four stranded β-sheet peptide-1. (A) Model helical peptide with a heme (engineered glycophorin A) and a β-barrel heme protein (PDB ID: 3CSN). (B) Design of peptide-1 with residues involved in heme ligation (red). (C) Section of a two-dimensional ^1^H–^1^H NOESY spectrum showing NOE connectivity between amide protons. Long range NOEs are underlined and boldfaced. (D) Superimposed twenty lowest energy structures of peptide-1. (E) Ribbon representation of the peptide-1 structure with side chains. (F) Heme binding isotherm of peptide-1.

Designing synthetic proteins displaying the structures and functions of naturally occurring proteins is of fundamental interest in chemistry and biology.^1^–^6^ Because of the diverse functions of heme *e.g.* enzymatic, electron transfer and energy conservation, designing heme-proteins has drawn considerable attention in the past and present years.^7^–^11^ It is worthwhile mentioning that the heme containing enzyme cytochrome P450 is vital for metabolizing over 75% of the drugs in the current market.^12^–^14^ Despite its high importance, the lack of an atomic-resolution structure of the membrane anchored full length cytochrome P450 impedes in-depth understating of enzyme's catalytic power.^12^,^13^ In particular, the structural plasticity of the catalytic site of cytochrome P450 remains elusive.^12^,^13^ Therefore, *de novo* designed heme binding peptides may be useful for the plausible dissection of complex catalytic mechanism. In nature, helical as well as β-sheet proteins are known to bind heme to carry out biological functions (Fig. 1A). *De novo* designing of heme-proteins and metallo-proteins using helical structural scaffolds has been highly successful.^7^–^11^,^15^–^17^ Recently, heme and metal binding helical proteins have been reported for the design of functional membrane proteins.^18^–^22^ Heme binding to helical proteins has been mostly achieved by taking advantage of the self-assembly of coiled–coiled structures. Here, either single or multiple heme groups can be intercalated, through appropriately placed His residues, at the interface of a coiled–coiled fold along the long axis of the helices (Fig. 1A). By contrast, the self-assembly of β-sheets or β-strands may often lead to heterogeneous aggregations or amyloid formations.^23^–^28^ The design of stable multi-stranded β-sheet structures or beta barrel structures soluble in a membrane environment could be even more complex, since the stability and folding characteristics of β-sheet membrane proteins are not currently well understood.^29^–^31^ Notably, *de novo* designed multi-stranded water soluble β-sheet proteins adopt open antiparallel β-sheet structures,^32^–^35^ with limited ligand binding sites. However, the design and characterization of atomic-resolution structures of β-sheet proteins that would bind to heme are yet to be reported. Beta barrel water soluble and membrane proteins are known to confer heme coordination.^36^,^37^ A heme-iron transporter beta barrel membrane protein, Has R, with 22 antiparallel beta-strands, binds heme at the surface of the barrel through bis-histidine coordination (Fig. 1A). In order to achieve heme binding β-sheet proteins, we have designed a series of peptides that would form well defined four stranded and six stranded β-sheet structures and display heme coordination at the targeted site with bis-histidine coordination. The designed peptides were solubilized, for functional and structural studies, in aqueous solutions containing dodecylphosphocholine providing a membrane mimic environment. Dodecylphosphocholine has been extensively used as a convenient detergent solution for reconstitution and NMR structure determination of membrane proteins including heme containing cytochromes.^38^–^41^ The sequences of peptide-1 to peptide-7 were designed to adopt a four stranded anti-parallel β-sheet structure where one heme cofactor is expected to be coordinated by two His residues between β-strand 2 and β-strand 3. Peptide-8 has been designed to adopt a six stranded β-sheet topology that would bind two molecules of heme, through bis-His coordination, between β-strand 2/β-strand 3 and β-strand 4/β-strand 5, respectively. The designed peptide sequences contain ^D^Pro-Gly residues for the correct juxtaposition among the anti-parallel β-strands nucleating either type I′ or type II′ β-turn conformations.^33^,^35^ Furthermore, the designed sequences are rich in aromatic and hydrophobic amino acids with a propensity to form β-structures. It may be noted that a myristoylated N-terminus sequence (IFW^D^PGHFV) used here for the β-sheet design has been previously investigated showing β-hairpin structure and low affinity binding to heme.^42^

## Results and discussion

### Topology and heme binding affinities of the designed four stranded β-sheet peptides

For the design of four stranded beta sheet peptides that would bind heme with high affinity at 1 : 1 stoichiometry, we first designed and investigated peptide-1 (Fig. 1B). The primary structure of peptide-1 contains three ^D^P-G sequences for the juxtaposition of the antiparallel β-strands in the four stranded β-sheet structure. The ^D^P9-G10 segment between strand 2 and strand 3 would bring residues H6 and H13 in close proximity to allow heme ligation. We have utilized NMR spectroscopy to determine the 3-D structures of the designed peptides. The secondary chemical shifts of the ^α^H of each residue of peptide-1 showed the existence of four β-strands as revealed from a positive deviation from the random coil values (ESI Fig. 1†). Residues ^D^Pro-Gly showed a negative deviation except for the residue ^D^P9. NOESY spectra of peptide-1 revealed diagnostic long-range cross-strand NOEs establishing a four β-sheet structure (Fig. 1C). An ensemble of 3-D structures of peptide-1 was obtained from medium and inter-strand long-range NOE-driven distance constraints (Fig. 1D and ESI Table 1†). The NMR structure of the designed peptide-1 is well defined with RMSD values for the backbone (C^α^, N, C′) atoms and all heavy atoms were estimated to be 0.30 Å and 0.57 Å, respectively (ESI Table 1†). ESI Table 1† summarizes the structural statistics of all the designed peptides. The four stranded β-sheet structure of peptide-1 is formed by strand 1: residues I1–W3, strand 2: residues H6–V8, strand 3: residues V11–H13 and strand 4: residues Y16–I18. The four stranded β-sheet topology of peptide-1 delineates close van der Waals packing interactions among the sidechains of residues with neighboring β-strands (Fig. 1D and E). Notably, packing interactions can be realized between residues F2/F7 in strand 1/strand 2, residues M12/I17 in strand 3/strand 4 and residues V8/V11 in strand 2/strand 3 (Fig. 1D and E). Moreover, heme coordinating residues H6 and H13 of peptide-1 are also found to be in close proximity (Fig. 1D and E). UV-vis spectroscopy experiments of the titration of peptide-1, monitoring the changes in the Soret band of heme, showed heme/peptide interactions (ESI Fig. 2A†). The heme binding isotherm of peptide-1 has been investigated by following the changes of heme absorbance at 412 nm (Fig. 1F). From analysis of the binding isotherm it can be seen that peptide-1 binds to heme with an apparent dissociation constant (*K*_d_) of 5.8 ± 0.7 μM (Table 1). A bis-histidine–heme ligation of peptide-1 was substantiated by the red shift of the Soret band to 428 nm upon dithionite reduction (ESI Fig. 2B†). The heme/peptide binding stoichiometry found by a Job's plot was 2 : 1 peptide : heme (ESI Fig. 2C†). Therefore, the data indicated that heme binding to peptide-1 plausibly requires a dimerization of the peptide in order to ligate one heme molecule through bis-histidine coordination. In other words, the structure and heme binding of peptide-1 revealed that the four stranded β-sheet structure is achieved as per the design, however, high affinity heme binding targeted to the interface between strand 2 and strand 3 of the four stranded β-sheet structure has not been successful. We envision that the low affinity heme binding and unexpected 2 : 1 stoichiometry observed for peptide-1 may have arisen due to the lack of a binding pocket for heme between strand 2 and strand 3. Thus, residue ^D^Pro9 was deleted in peptide-2 which may essentially diminish the packing interactions between strand 2 and strand 3. However, peptide-2 also showed a 2 : 1 (peptide : heme) stoichiometric ratio and an even lower *K*_d_ value of 8.9 ± 0.9 μM for heme binding (Table 1). NMR analyses and atomic-resolution structure revealed that peptide-2 adopts a four stranded β-sheet structure with a hinge at residue G9, however, the packing interactions between strand 2 and strand 3 have been highly diminished (ESI Fig. 3†).

**Table 1:** Summary of heme binding affinity and enzymatic efficacy of the designed peptides^*a*^

Peptide	*K*_d_ μM	*k*_cat_/*K*_m_ × 10^7^ M^–1^ s^–1^
1	5.8 ± 0.7	2.84
2	8.9 ± 0.9	2.91
3	2.7 ± 0.5	2.79
4	2.0 ± 0.2	2.44
5	1.6 ± 0.2	1.57
6	0.8 ± 0.2	1.34
7	0.4 ± 0.2	1.15
8	0.5 ± 0.1	4.35
9	28.1 ± 3.2	2.21
10	19.5 ± 2.1	1.30

^*a*^9 and 10 represent peptide-8H6AH12A and peptide-8H15AH21A, respectively.

**Fig. 2:**
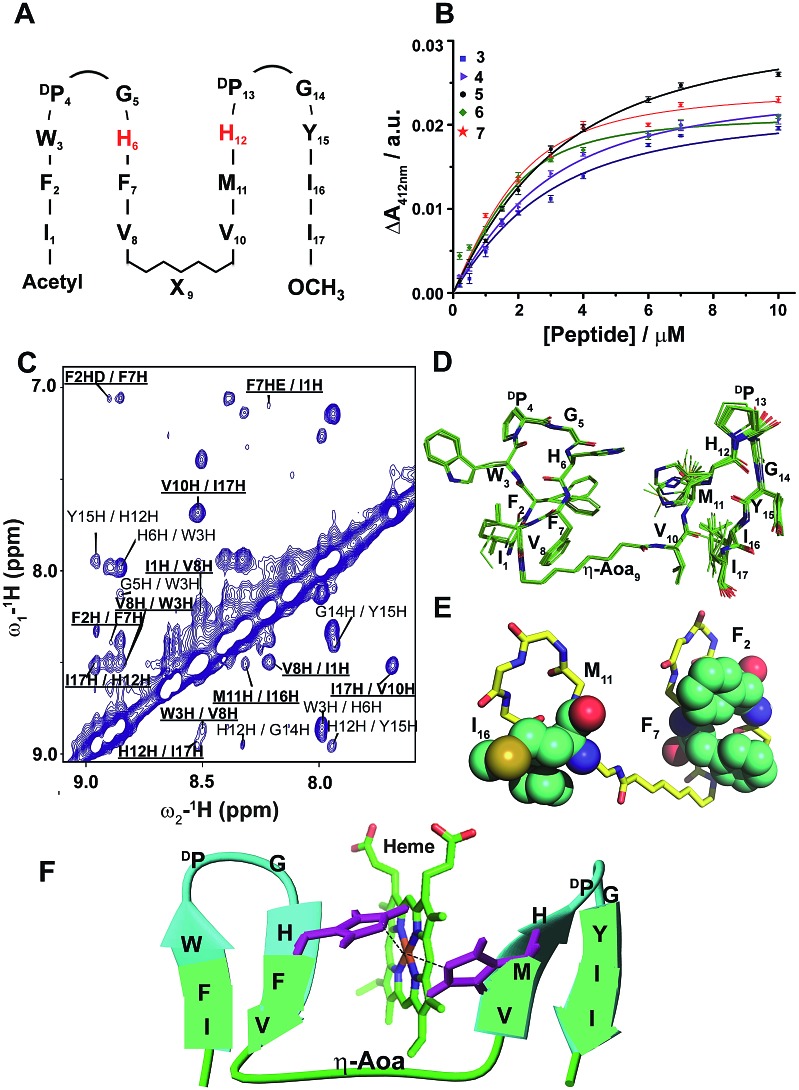
Design, structure and heme binding of four stranded β-sheet peptides. (A) Design of peptides 2–7 with the His residues involved in heme ligation shown in red. X_9_ denotes Gly, βAla, δAva, εAca, ζAha and ηAoa in peptides 2, 3, 4, 5, 6 and 7, respectively. (B) Heme binding isotherms of peptides 3 (blue), 4 (purple), 5 (orange), 6 (green) and 7 (red). (C) Section of two-dimensional ^1^H–^1^H NOESY spectrum showing NOE connectivity between amide protons. Long range NOEs are underlined and boldfaced. (D) Superimposed twenty lowest energy structures of peptide-7. (E) A selected structure of peptide-7 showing side chain packing within β-sheets. (F) Model structure of a heme–peptide-7 complex. The side chains of the two axial ligands coordinating heme are shown (purple). Residues localized at the micelle/water interface (cyan) and residues localized in the hydrophobic core of micelles (green) are highlighted.

In order to create a high affinity heme binding pocket, we have replaced the ^D^P9-G10 sequence motif with amino acid β-Ala, in peptide-3. We assume that a higher degree of freedom at the Cβ–Cα bond of β-Ala^43^–^45^ may allow one heme moiety to be coordinated with two His residues in the four stranded β-sheet scaffold. Interestingly, peptide-3 binds heme with significantly lowered *K*_d_, 2.7 ± 0.5 μM, compared to peptide-1 and peptide-2 with an expected stoichiometry of a peptide : heme 1 : 1 ratio (Fig. 2A and B, Table 1, ESI Fig. 4†). A bis-histidine heme coordination of peptide-3 has been confirmed by heme reduction upon addition of sodium dithionite (ESI Fig. 4†). Secondary chemical shifts of ^α^Hs showing positive deviation indicated four β-strands (ESI Fig. 5A†) and was further supported by several cross-strand NOEs for peptide-3 (ESI Fig. 5B†). NMR analysis revealed a four stranded β-sheet structure of peptide-3 with limited packing interactions between strand 2 and strand 3 (ESI Fig. 5C†). The β-Ala residue assumed an extended conformation and appeared to be forming a short loop between strand 2 and strand 3 (ESI Fig. 5C†). The NMR structures of peptide-3 and peptide-2 demonstrated that the heme coordinating His residues are not in close proximity in their apo-forms. However, it is likely that the loop flexibility conferred by the single methylene bridge between CH_2_–CH_2_ groups of β-Ala, compared to Gly, enables bis-histidine heme ligation with H6 (in strand 2) and H12 (in strand 3) of peptide-3. Encouraged by these observations, we further systematically investigated the length of the methylene units with heme binding affinity and stoichiometry. Designed peptides 4 to 7 contain an increasing number of methylene units from δ-aminovaleric acid (three methylene groups) in peptide-4, ε-aminocaproic acid (four methylene groups) in peptide-5, and ζ-aminoheptanoic acid (five methylene groups) in peptide-6, to η-aminooctanoic acid (six methylene groups) in peptide-7. Heme binding studies of these peptides demonstrated that there was a progressive increase in heme binding affinity with the increase of the number of methylene units (Table 1). Heme binds to peptide-7 with the lowest *K*_d_ value, of 400 nM, among the designed peptides, and with a 1 : 1 molar ratio (Fig. 2B, ESI Fig. 6†). Secondary chemical shifts and NMR structures of peptides 4, 5 and 6 have been provided in ESI Fig. 7 and 8† establishing their four stranded β-sheet topology.

Due to its high affinity binding to heme, structure and micelle insertion, investigation of peptide-7 has been further carried out in more detail. CD spectroscopy has been used to determine the global conformations of peptide 7 in apo and holo states (ESI Fig. 9†). Peptide-7 showed an intense negative CD band at ∼212 nm and a relatively low intense positive CD band at ∼225–230 nm (ESI Fig. 9†). These CD bands are diagnostic of β-sheet secondary structures containing exciton coupling arising from packing involving aromatic sidechains. The CD spectra of peptide-7 in complex with heme showed discernable changes (ESI Fig. 9†). As seen, the far UV CD band of the holo peptide-7 has been shifted from 212 nm (apo peptide) to 216 nm with a more intense band at 230 nm. These spectral changes might be reflecting an enhancement of the sidechain–sidechain packing interactions of the heme-bound peptide. Heme in complex with the designed peptide-7 showed an induced CD band in the Soret region of the absorption spectra, indicating that the heme is experiencing a chiral environment in the holo state (ESI Fig. 9†). Secondary chemical shifts of ^α^Hs and inter-strand NOEs of peptide-7 were used to identify segments of four β-strands and two β-turns for ^D^P-G sequences (ESI Fig. 7†). Fig. 2C shows a section of a NOESY spectrum showing sequential and cross-strand backbone NH/NH NOEs of peptide-7. The NMR determined structure of peptide-7 has been further refined using paramagnetic (PRE) NMR driven distance constraints since cross-strand NOEs were in paucity among the residues of strand 2 and strand 3 limiting the resolution of their spatial proximity in the structure. Residue H6 (in β-strand 2) of peptide-7 was replaced by residue Cys and labelled with MTSL, a paramagnetic probe (on line methods) that would enhance the relaxation of the NMR active nuclei within a 20 Å distance. The intensity changes of NH/C^α^H cross-peaks observed in 2-D TOCSY spectra due to the PRE effect were utilized as distance constraints for the structure calculations for peptide-7 (ESI Table 1†). The 3-D structure of peptide-7 reveals that strand 2 and strand 3 are orientated in a proximal fashion with a plausible binding pocket for heme insertion and ligation (Fig. 2D and E). Localization of peptide-7 into DPC micelles was investigated by PRE mediated resonance perturbation using spin labelled lipid 16-doxyl stearic acid (16-DSA). The PRE probe, 16-DSA, would perturb the resonances of amino acids located in the hydrophobic environment of a micelle, whereas residues at the micelle/water interface would experience a lower degree of change. 2-D TOCSY spectra of peptide-7 were obtained either in the absence of 16-DSA or in the presence of 16-DSA and the backbone C^α^H/NH cross-peak intensity changes were analyzed (ESI Fig. 10†). Residues I1, F2, V8, η-aminooctanoic acid, V10, M11, Y15, I16 and I17 were significantly perturbed by the inclusion of 16-DSA whereas the residues of β-turns were less affected by the PRE probe (Fig. 2F, ESI Fig. 10†). These observations indicated that hydrophobic residues in the four β-strands including η-aminooctanoic acid of peptide-7 are well inserted into the non-polar environment of micelles whereas residues in the β-turns including residues H6 and H12 are localized at the water/micelle interface. The surface localization of residues H6 and H12 provides coordination with the cofactor heme and may help in carrying out interfacial functions. The 3-D structure of a heme–peptide complex is not yet available due to the difficulty in obtaining NMR spectra for the heme–peptide complex resulting from the high pH and high detergent concentrations required for NMR studies of a heme–peptide complex. However, a molecular model of the heme/peptide-7 complex (Fig. 2F) shows a plausible mode of heme insertion at the binding pocket between strand 2 and strand 3 of peptide-7.

### Topology and heme binding affinity of di-heme binding six stranded β-sheet peptides

**Fig. 3:**
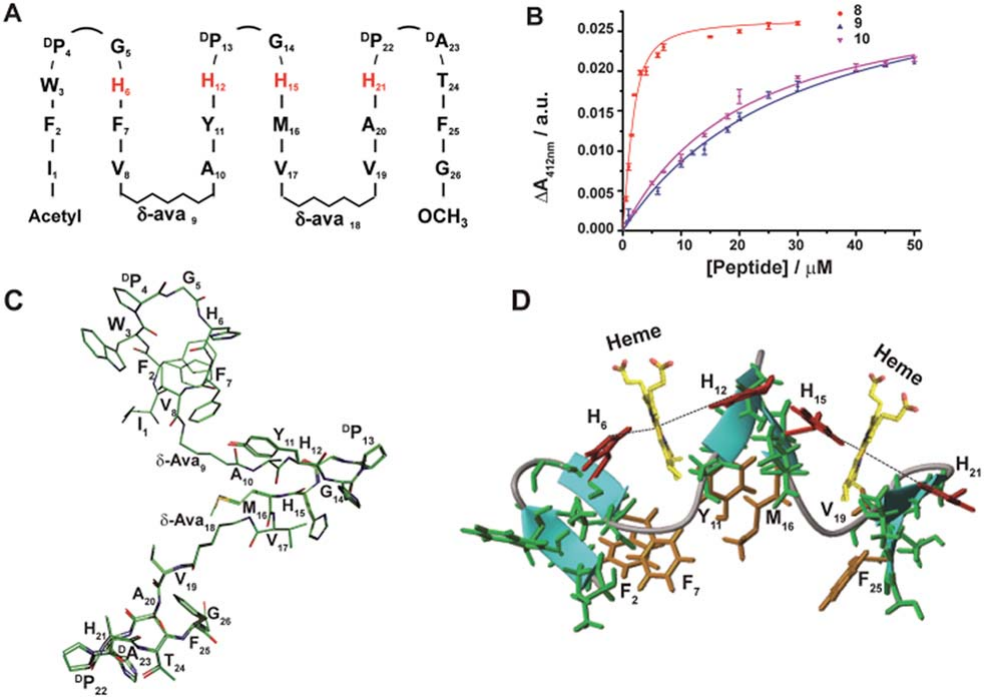
Design, structure and heme binding of six stranded β-sheet peptides. (A) Design of peptide-8 with His residues involved in heme ligation are shown in red. (B) Heme binding isotherms of peptide-8 (red), peptide-8H6AH12A or 9 (blue) and peptide-8H15AH21A or 10 (pink). (C) Superimposed ten lowest energy structures of peptide-8. (D) Model structure of the heme–peptide-8 complex. The side chains of the four axial ligands coordinating heme are shown (red). Residues involved in side chain packing within β-sheets are highlighted (gold).

We extended the design strategy to construct 26-residue six stranded β-sheet peptides that would ligate two heme molecules, employing residues H6/H12 at strand 2/strand 3 and residues H15/H21 at strand 4/strand 5, respectively (Fig. 3A). We used δ-aminovaleric acid between strand 2 and strand 3, and strand 4 and strand 5 to accommodate two heme molecules in the designed structure. Diagnostic inter-strand NOEs pertaining to a β-sheet structure were detected for peptide-8 (ESI Fig. 11†). An ensemble of structures for peptide-8 has been determined based on NOE-driven distance constraints (Fig. 3C, ESI Table 1†). An NMR structure of peptide-8 confirms a six stranded β-sheet topology with loop conformations for the δ-aminovaleric acid residues (Fig. 3D). The six stranded β-sheet structure of peptide-8 is typified by sidechain–sidechain packing among residues F2/F7, Y11/M16, and V19/F25 (Fig. 3D). The heme binding of peptide-8 was estimated from the spectral changes of the Soret band upon addition of peptide-8 (ESI Fig. 12A†). Peptide-8 binds to heme in a 2 : 1 (heme : peptide) stoichiometry as obtained from a Job's plot (ESI Fig. 12B†). Fig. 3B shows the heme binding isotherm of peptide-8 delineating a sharp increase followed by saturation at higher concentrations of peptide-8. Binding isotherms revealed that peptide-8 binds heme with a *K*_d_ of 0.5 ± 0.1 μM (Table 1). Dithionite reduction of heme absorption spectra indicated a bis-histidine coordination of peptide-8 (ESI Fig. 12C†). The 3-D structure of peptide-8 in complex with two heme molecules has yet to be determined; however it is highly likely that the binding of two heme molecules to the apo-peptide may cause a facile structural rearrangement defining the orientation of heme binding β-strands (Fig. 3D).

We note that peptide-8 demonstrated an appreciably lower *K*_d_ compared to peptide-4, a four stranded β-sheet containing δ-aminovaleric acid (Table 1). The heme binding isotherm and lower *K*_d_ value of peptide-8 potentially indicated plausible cooperative heme interactions with peptide-8. A dramatic decrease in the heme binding affinity was observed for analog 8H15AH21A, binding heme between strand 2 and strand 3, and an appreciably higher *K*_d_ of 19.5 μM was displayed (Table 1). On the other hand, even a somewhat higher *K*_d_ value of 28 μM, was estimated for peptide-8H6AH12A (Table 1). These data indicated that two molecules of heme bind to peptide-8 in a highly cooperative manner, whereas the ligation of a single heme is highly impaired. The origin of such cooperativity is likely involve the rearrangement of β-strands at the heme binding pockets upon ligation of two heme moieties.

### Peroxidase activity of the designed β-sheet peptides

**Fig. 4:**
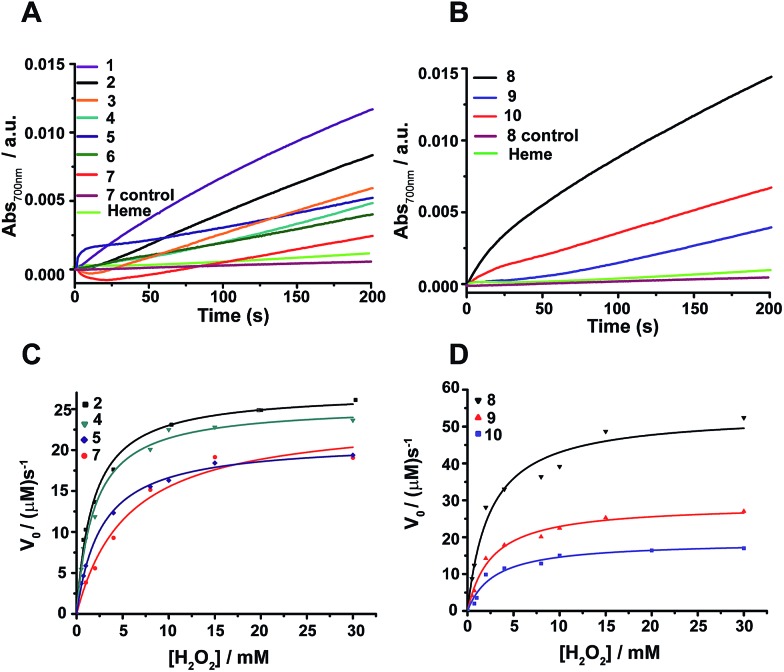
Peroxidase activity of designed β-sheet peptides. (A) Time course of ABTS oxidation at 700 nm for four stranded peptide–heme complexes at 4 mM H_2_O_2_ concentration. (B) Absorbance of ABTS oxidation at 700 nm *versus* time for six stranded peptide–heme complexes at 4 mM H_2_O_2_ concentration. (C) Steady-state kinetics of ABTS oxidation as a function of hydrogen peroxide concentration for peptides 2, 4, 5 and 7. (D) Steady-state kinetics of ABTS oxidation as a function of hydrogen peroxide concentration for peptides 8, 9 and 10. In all cases, the final heme, peptide and ABTS concentration were 0.5 μM, 4 μM and 2.5 mM respectively in sodium phosphate buffer, pH 7.2 containing 2 mM DPC. The data was fitted to a Michaelis–Menten equation to derive catalytic parameters (Table 1).

The peroxidase activity of heme bound peptides was examined using 2,2′-azino-bis(3-ethylbenzthiazoline-6-sulfonic acid) (ABTS), a substrate for peroxidase studies that undergoes a single-electron oxidation, as a reducing agent. Fig. 4A and B show the time course of oxidation of ABTS in the presence of H_2_O_2_ for the four stranded β-sheet peptides (Fig. 4A) and the six stranded β-sheet peptide-8 and mutants in complexes with heme (Fig. 4B). Enzymatic activity studies were also carried out for free peptides and also for free heme (Fig. 4A and B). These data suggest that peroxidase activity can be detected for heme–peptide complexes, although with different kinetics. Free heme or apo-peptides do not possess any enzymatic activity. Furthermore, the changes of the initial rates of product formation as a function of hydrogen peroxide concentrations for four stranded (Fig. 4C) and six stranded peptides (Fig. 4D) were estimated. Enzyme activity data were analyzed using the Michaelis–Menten equation yielding *k*_cat_ and *K*_M_ parameters (Table 1). The catalytic efficiency, *k*_cat_/*K*_M_, among four stranded β-sheet peptides, peptide-1 to peptide-7, shows significant variation (Table 1). Peptide-2 demonstrated a high catalytic efficiency with a *k*_cat_/*K*_M_ of 2.91 × 10^7^ M^–1^ s^–1^, whereas the catalytic efficiency of peptide-7 was estimated to be the lowest with a *k*_cat_/*K*_M_ value of 1.15 × 10^7^ M^–1^ s^–1^ (Table 1). The peroxidase activity of the four stranded β-sheet peptides appears to follow an inverse correlation with their heme binding affinity. In other words, with the tightest heme binding, peptide-7 showed the lowest catalytic activity, whereas peptide-2 with the lowest heme affinity showed the highest peroxidase activity (Table 1). As observed in heme-proteins, a tighter heme binding would confer a more rigid axial position of the porphyrin ring by coordinating the histidine residue that reduces susceptibility to hydrogen peroxide.^43^,^46^ The peroxidase activity of peptide-8, the six stranded β-sheet peptide, is remarkably high compared to peptide-2 with a *k*_cat_/*K*_M_ of 4.35 × 10^7^ M^–1^ s^–1^ (Table 1). The superior peroxidase activity of peptide-8 may be attributed to the presence of two heme moieties involved in catalysis. The mutant peptides, peptide-8H15AH21A and peptide-8H6AH12A, were appreciably less active compared to the parent peptide with lower *k*_cat_/*K*_M_ values (Table 1). The catalytic efficiency of the mutant peptides appears to be comparable to the four stranded β-sheet peptides binding to a single heme. It may be noted that the peroxidase activity of the designed peptides was significantly lower in comparison to the naturally occurring peroxidases^9^,^19^,^46^ possibly due to the hexa-coordinated states of heme. However, the designed peptides displayed peroxidase activity akin to previously reported designed heme proteins and peptides.^9^,^19^,^46^

### Electron transfer activity of peptide-7 and peptide-8 with cytochrome c

**Fig. 5:**
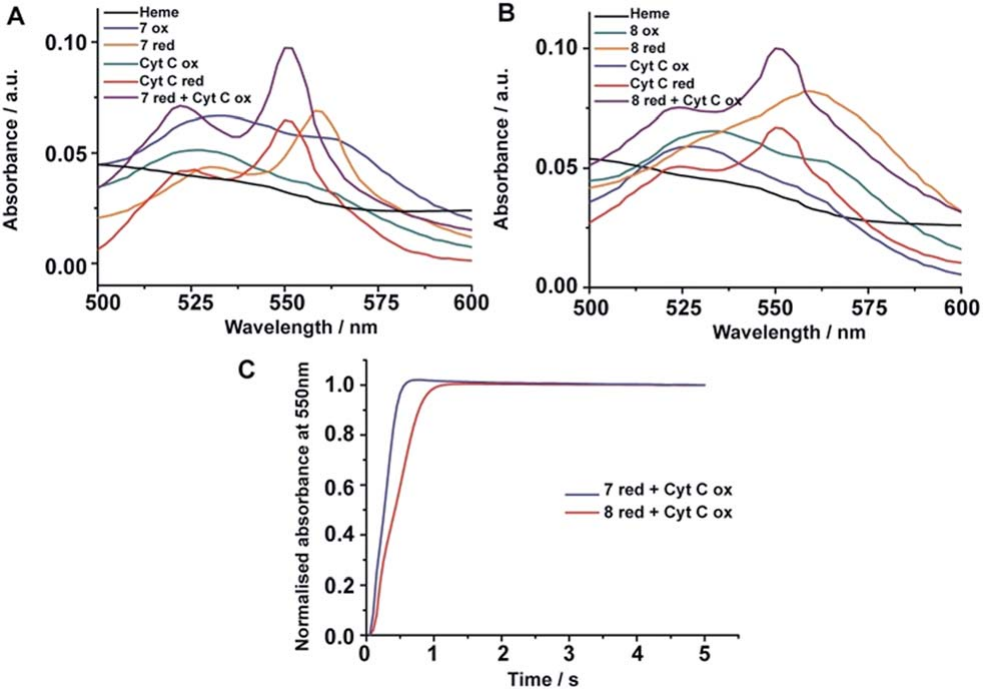
Electron transfer of four stranded and six stranded β-sheet peptides with cyt c. (A) Absorption spectra of heme alone (black); peptide 7-heme: oxidized (blue), reduced (orange); cyt c: oxidized (green), reduced (red); and 2 : 1 equivalent mixture of reduced peptide 7-heme and oxidized cyt c (purple). (B) Absorption spectra of heme alone (black); peptide 8-heme: oxidized (green), reduced (orange); cyt c: oxidized (blue), reduced (red); and 2 : 1 equivalent mixture of reduced peptide 8-heme and oxidized cyt c (purple). (C) Absorbance monitored at 550 nm *versus* time for reduced peptide 7-heme/cyt c oxidized (blue) and reduced peptide 8-heme/cyt c oxidized (red). The peptide/heme concentration was 24 μM and cytochrome c concentration was 12 μM.

Heme containing proteins carry out electron transfer processes with other proteins in cell energy production. We investigated the electron transfer activity of peptide-7 and peptide-8 with cytochrome c (cyt c), a small heme protein residing in the inner membrane space of mitochondria and known to be associated with lipids and micelles. Cyt c is a component of the electron transfer chain in mitochondria required for the production of ATP. The heme group of cyt c performs electron transfer with membrane proteins bc1 complex and complex 4.^47^Fig. 5A compares the UV-vis absorption spectra (500–650 nm) of heme in complex with peptide-7, cyt c in oxidized and also in reduced states and a 2 : 1 mixture of peptide-7 (reduced) and cyt c (oxidized). The reduced heme in peptide-7 showed two absorption peaks at 530 and 560 nm, whereas the reduced heme of cyt c displayed absorption peaks in different positions at 520 and 550 nm (Fig. 5A). The absorption maxima of the oxidized heme for both peptide-7 and cyt c were characterized by broad and unresolved peaks (Fig. 5A). Interestingly, the absorption spectra of heme in the mixture of reduced heme/peptide-7 complex and oxidized heme of cyt c revealed absorption spectra corresponding to reduced heme of cyt c (Fig. 5A). Similar observations can be made from the heme absorption spectra of peptide-8 and cyt c (Fig. 5B). These data clearly demonstrated that the designed heme–peptides are able to undergo electron transfer reactions with a naturally occurring protein involved in the electron transport chain. The kinetics of electron transfer reactions were further examined for the reduced heme–peptides, peptide-7 and peptide-8, and oxidized cyt c following changes in absorption at a 550 nm wavelength (Fig. 5C). The rate constant of electron transfer has been found to be 2.28 s^–1^ and 1.6 s^–1^ for peptide-7 and peptide-8, respectively. Peptide-7, containing a single heme, shows an approximately 1.4 times faster electron transfer rate compared to the di-heme binding peptide-8. It may be noted that electron transfer rate between cyt c and cytochrome c peroxidase has been estimated to be 0.23 s^–1^.^48^ These studies indicated that an efficient electron transfer occurs between peptide-7 and peptide-8 with cyt c.

## Conclusions

Emulation of structures and functions of large proteins in small peptides is a challenging task. Unlike proteins, small peptides often lack defined folded structures and binding surfaces for interactions with ligands. Coiled coil helical peptides can self-assemble capturing heme within its binding interfaces. However, self-assembled β-sheet structures often generate insoluble fibrils or amyloid-like materials. The present work demonstrates novel design principles for developing high affinity heme binding to well-defined multi-stranded β-sheet peptides. The introduction of beta and omega amino acids as loop residues in designing a high affinity heme binding pocket in β-sheet structures is critical. The designed peptides display enzymatic activity and facilitate electron transfer with a protein in an electron transport chain in a membrane mimetic environment. These structures and designs can be further utilized to generate new classes of heme and/or metallo-peptides and proteins functional in membrane and in solution. In addition, the binding of heme to β-sheet structures has implications in understanding the mechanism of amyloid formation and neurotoxicity in Alzheimer's disease as heme/a-beta complexes demonstrate peroxidase activity and have been linked to cellular neurotoxicity and aberrant neurotransmission.^49^ Designed β-sheet/heme complexes would perhaps serve as model systems towards the understanding of the molecular mechanism of the neurotoxicity of amyloids. Furthermore, autonomously folded synthetic β-sheet peptides in a membrane environment may be exploited for destabilizing membrane protein/protein interactions for the development of novel therapeutics.^50^

## Materials and methods

### Peptides and materials

Peptides were commercially synthesized from GL-Biochem (Shanghai, China). Crude peptides were subjected to purification by reverse phase HPLC Waters™ using a C_4_ column (300 Å pore size, 5 μM particle size). A linear gradient of acetonitrile/water (both solutions containing 0.1% v/v TFA) was used to elute the peptides while maintaining a constant flow rate of 2 mL min^–1^. The major sharp peak fraction obtained was then pooled and lyophilized. The masses of the peptides were confirmed by mass spectrometry. DPC and deuterated compounds (DPC-d_38_, D_2_O) were purchased from Avanti polar lipids (Alabama, USA) and Cambridge Isotope Laboratories Inc. (Massachusetts, USA), respectively. 16-DSA was obtained from Sigma (St. Louis, MO, USA). 4,4-Dimethyl-4-silapentane-1-sulfonic acid (DSS) was acquired from Cambridge Isotope Laboratories Inc. (Massachusetts, USA). Other chemicals including sodium dithionite, hemin, ABTS, and cytochrome c were of analytical grade and purchased from Sigma-Aldrich.

### Heme binding and stoichiometric analysis

The binding affinity of heme to the designed peptides was characterized using a multi-well plate reader (Tecan Infinite M200 PRO) by titrating increasing concentrations of peptide (0–30 μM) into a solution of 2 μM heme prepared in 50 mM sodium phosphate buffer, 2 mM DPC, pH 7.2. The Soret band at 412 nm was monitored for complex formation for each aliquot after three hours of incubation. Wavelength scans from 350–600 nm were also recorded to monitor the shift in the absorption maxima of heme on binding to the peptides. Binding isotherms were obtained by plotting the absorbance at 412 nm *versus* the peptide concentration. The values of Δ*ε* and *K*_d,app_ were derived from the ligand depletion binding model fit using Origin 9.0.^16^,^51^ 
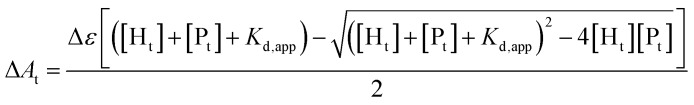
[H_t_] is the total heme concentration, [P_t_] is the total peptide concentration, *K*_d,app_ is the apparent dissociation constant, Δ*ε* is the difference in extinction coefficients of bound and unbound heme, and Δ*A*_t_ is the change in absorbance for a fixed peptide concentration.

Peptide–heme stoichiometry was determined using a method of continuous variation or Job's plot. 50 μM peptide and heme stocks were prepared in 2 mM DPC, 50 mM sodium phosphate buffer, pH 7.2. Different ratios of the two stock solutions were mixed while keeping the total volume constant. The absorbance values at 412 and 356 nm were recorded for the various mole fractions of heme using a multi-well plate reader (Tecan Infinite M200 PRO). This difference in absorbance was then plotted against the mole fraction of heme.

### Electron transfer experiment

A heme–peptide sample was prepared in 2 mM DPC, 50 mM sodium phosphate buffer, pH 7.2 by incubating 24 μM peptide with 24 μM heme for 3 h. Reduced heme–peptide was prepared by the addition of sodium dithionite to the heme–peptide sample. Cytochrome c of concentration 12 μM was prepared using identical buffer conditions. Absorbance scans were monitored from 350–600 nm in a multi-well plate reader (Tecan Infinite M200 PRO). The kinetics of the electron transfer reaction was followed spectrophotometrically at room temperature using stopped-flow kinetics apparatus (SX20, Applied Photophysics). The rate constant for the reaction was determined by monitoring the changes in the absorption intensity at 550 nm *versus* time.

### Enzymatic assays

Peroxidase activity was evaluated in the presence of co-substrate ABTS, using stopped-flow apparatus (SX20, Applied Photophysics). While one syringe was loaded with 8 μM peptide, 1 μM heme and 5.0 mM ABTS mixture in 2 mM DPC sodium phosphate buffer, pH 7.2, the other syringe was loaded with hydrogen peroxide of required concentration in identical buffer. The hydrogen peroxide stock solution concentration was standardized by UV-vis measurements (*ε*_230_ = 72.8 M^–1^ cm^–1^). The reaction was initiated by injection of equal volumes from both syringes. The final heme, peptide, and ABTS concentrations were 0.5 μM, 4 μM and 2.5 mM, respectively for each reaction. The steady-state kinetic parameters (*K*_m_, *V*_max_) in the presence of ABTS were quantified by varying final concentrations of H_2_O_2_ from 0.5–30 mM and monitoring the absorbance at 700 nm. The absorbance at 700 nm was then converted to concentration (*ε*_700 nm_ = 1.6 × 10^4^ M^–1^ cm^–1^). The reaction rates (*V*_0_) for reactions at different H_2_O_2_ concentrations were determined by linear regression analysis. The initial rates *vs.* H_2_O_2_ concentration were plotted and fitted to the Michaelis–Menten equation using Origin 9.0 software.
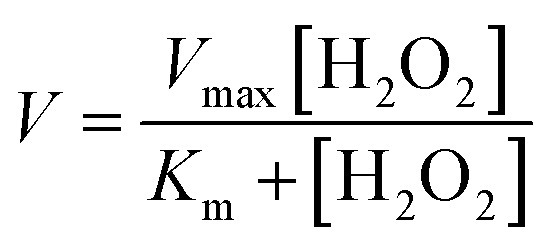
 *V* is the reaction rate (μM s^–1^), *V*_max_ is the maximal velocity for the enzymatic reaction (μM s^–1^), *K*_m_ is the Michaelis–Menten constant and [H_2_O_2_] is the concentration of H_2_O_2_ used. Turnover numbers (*k*_cat_) were calculated by dividing the maximal velocity by the concentration of heme–peptide complex.

### NMR and structural characterization

All NMR experiments were performed at 42 °C on a Bruker DRX 600-MHz spectrometer equipped with a cryo-probe. NMR samples were prepared by dissolving the lyophilized peptide in 500 μL 90% H_2_O/10% D_2_O containing 125 mM per-deuterated DPC. The concentration of the NMR sample was approximately 0.3–0.5 mM. Two dimensional TOCSY (mixing time, 80 ms) and NOESY (mixing time, 200 ms) experiments were acquired for spin system assignments and structure determination. The chemical shifts of protons were internally referenced to 4,4-dimethyl-4-silapentane-1-sulfonic acid (DSS). The proton resonances of all the amino acids were assigned unambiguously by overlaying TOCSY and NOESY spectra and sequential walking. Backbone dihedral angles were then predicted after obtaining Hα, an amide proton, (and ^13^Cα chemical shifts) of the assigned NOESY and ^13^C–^1^H HSQC spectra using PREDATOR. NOE restraints were derived from the NOE intensities of the assigned NOESY spectra and categorized into strong, medium and weak, respectively. This was further translated to distance limits between 2.5 and 5.0 Å. The NOE restraints and predicted dihedral angle values were used to carry out several rounds of structure calculations using CYANA 2.1. Of the 100 structures generated, 20 energy minimized structures were selected for evaluation and analysis. PROCHECK-NMR was used to assess the stereo-chemical quality of the structure ensembles. The structures were analyzed visually by PYMOL and MOLMOL. PRE experiments were carried out by titrating 2 mM 16-doxyl-stearic acid (16-DSA) dissolved in deuterated methanol to a lyophilized peptide sample in DPC micelles. Two dimensional TOCSY spectra were carried out with and without the PRE probe using the same experimental conditions. The intensities of the CαH/NH cross peaks were evaluated before and after the addition of the PRE probe and their ratios plotted for each amino acid. To facilitate MTSL (*S*-(1-oxyl-2,2,5,5-tetramethyl-2,5-dihydro-1*H*-pyrrol-3-yl)methyl methanesulfonothioate) labelling, peptide-7H6C was incubated with 10 : 1 MTSL : peptide concentration in 50% acetonitrile solution and incubated overnight at room temperature (>12 hours). In-order to remove excess MTSL and unlabelled peptide, the incubated sample was diluted and subjected to HPLC purification. The major eluted peaks were analyzed by mass spectrometry and the labeled peptide fraction was verified. The labeled peptide was then reconstituted in identical buffer conditions and two dimensional TOCSY spectra were carried out for the labelled and unlabelled peptide. The intensity of the TOCSY peaks observed for the labelled sample were used as a measure to derive distance restraints for the peptide-7 structure.

## Supplementary Material

Supplementary information
