# Correction: Designed multi-stranded heme binding β-sheet peptides in membrane

**DOI:** 10.1039/c6sc90078j

**Published:** 2016-11-22

**Authors:** Areetha D'Souza, Mukesh Mahajan, Surajit Bhattacharjya

**Affiliations:** a School of Biological Sciences , 60 Nanyang Drive , 637551 , Singapore . Email: surajit@ntu.edu.sg ; Fax: +65-6791-3856

## Abstract

Correction for ‘Designed multi-stranded heme binding β-sheet peptides in membrane’ by Areetha D'Souza *et al.*, *Chem. Sci.*, 2016, **7**, 2563–2571.



## 

There has been a mistake in the nomenclature of the non-natural amino acids used in the peptide design. The sentence “Designed peptides **4** to **7** contain increasing number of methylene units from δ-aminovaleric acid (three methylene groups) in peptide-**4**, ε-aminocaproic acid (four methylene groups) in peptide-**5**, ζ-aminoheptanoic acid (five methylene groups) in peptide-**6**, η-aminooctanoic acid (six methylene groups) in peptide-**7**” should be corrected to the following: “Designed peptides **4** to **7** contain increasing number of methylene units from δ-aminovaleric acid (four methylene groups) in peptide-**4**, ε-aminocaproic acid (five methylene groups) in peptide-**5**, ζ-aminoheptanoic acid (six methylene groups) in peptide-**6**, η-aminooctanoic acid (seven methylene groups) in peptide-**7**”.

The Royal Society of Chemistry apologises for these errors and any consequent inconvenience to authors and readers.

