# Supplementary material for: Designed multi-stranded heme binding β-sheet peptides in membrane
Source: Chem Sci. 2015 Dec 17;7(4):2563–71. doi: 10.1039/c5sc04108b (PMC5477022; doi:10.1039/c5sc04108b)
Supplement: Supplementary file 1 [file SC-007-C5SC04108B-s001.pdf]

Electronic Supplementary Material (ESI) for Chemical Science.
This journal is © The Royal Society of Chemistry 2015

**Supplementary Results**

# Designed Multi-stranded Heme Binding β-Sheet Peptides in Membrane

Areetha D'Souza, Mukesh Mahajan and Surajit Bhattacharjya*

School of Biological Sciences, Structural Biology and Biochemistry, Nanyang Technological University, Singapore 637551.

*Address correspondence to: Surajit Bhattacharjya, School of Biological Sciences, 60 Nanyang Drive, Singapore, 637551, e-mail: surajit@ntu.edu.sg, Fax: 65-6791-3856

**Figure S1**

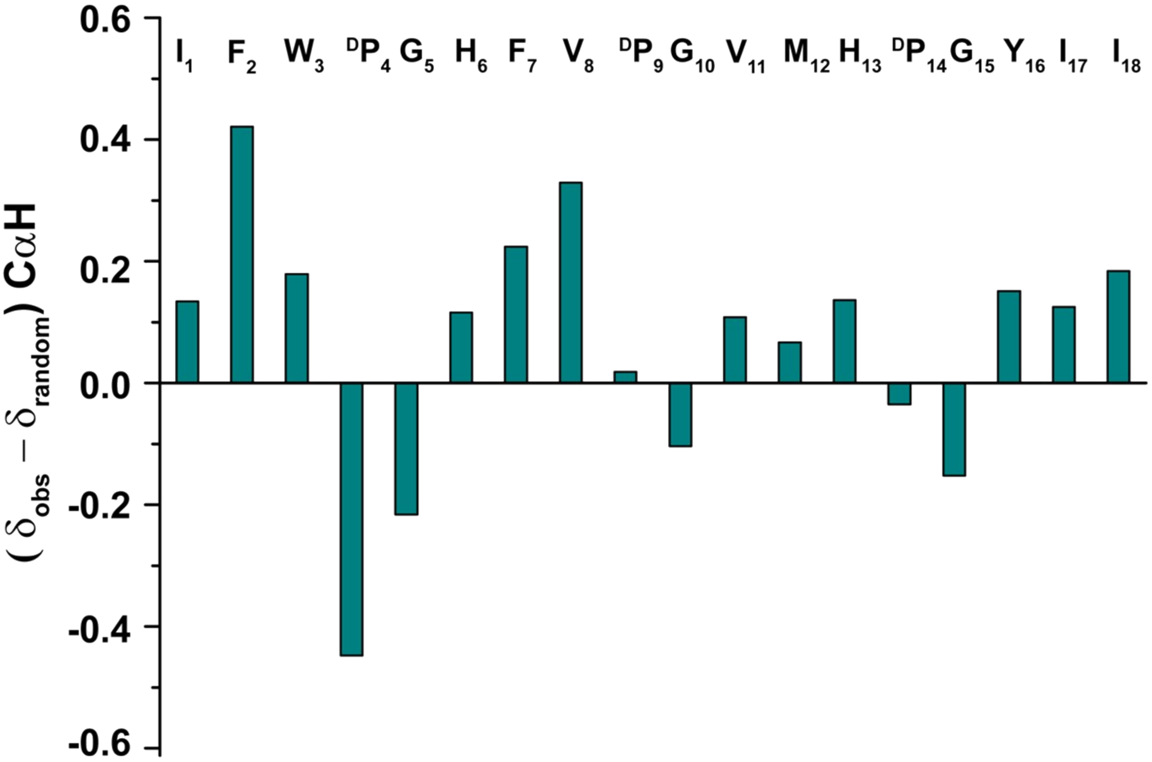


**Supplementary Figure 1:** αH chemical shift deviation from random coil values of peptide-1. The positive deviation of residues I1, F2, W3, H6, F7, V8, V11, M12, H13, Y16, I17 and I18 indicates four stranded β-sheet structure

**Figure S2**

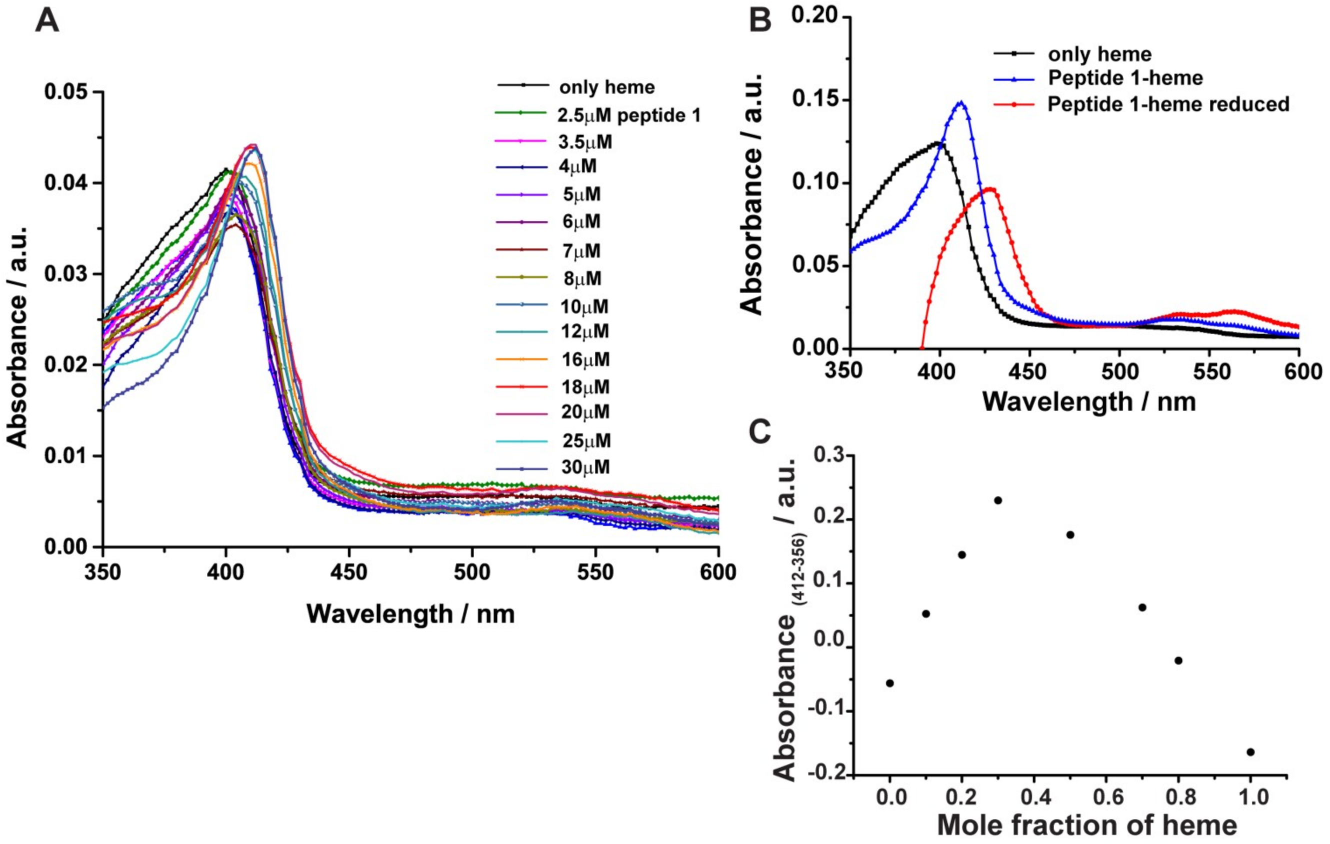


**Supplementary Figure 2:** (A) Absorption changes of heme (2 μM) on titrating peptide-1(0-30 μM) in 50 mM sodium phosphate buffer, pH 7.2 containing 2 mM DPC. (B) Absorption spectra of heme alone (black), peptide1-heme oxidized (blue) and peptide1-heme reduced (red). Concentration of peptide and heme was 10 μM in 2 mM DPC, 50 mM sodium phosphate buffer, pH 7.2 (C) Jobs plot of peptide-1. The shift in Soret band of heme at 412 nm upon binding to peptide-1 and further shift to 428 nm after reduction by sodium dithionite and low intense peaks at 530 and 560 nm indicated bis-histidine coordination. The job plot shows peptide:heme stoichiometry 2:1

**Figure S3**

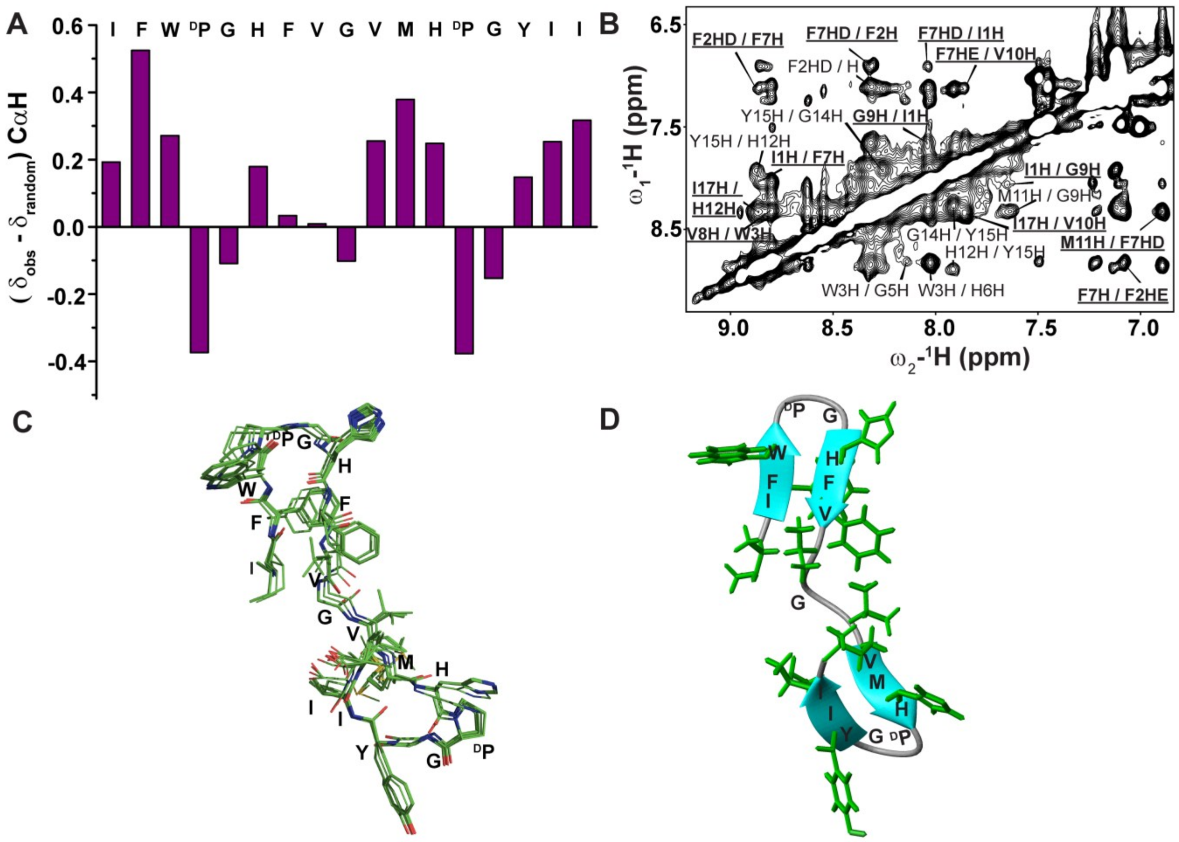


**Supplementary Figure 3:** (A) αH chemical shift deviation from random coil of peptide-2 (B) Section of two-dimensional $^1H$-$^1H$ NOESY spectra showing NOE connectivity between amide protons. Long range NOEs are underlined and boldfaced. (C) Superimposed twenty low energy structures of peptide-2. (D) A selected structure of peptide-2 showing side chain packing within β-sheets.

**Figure S4**

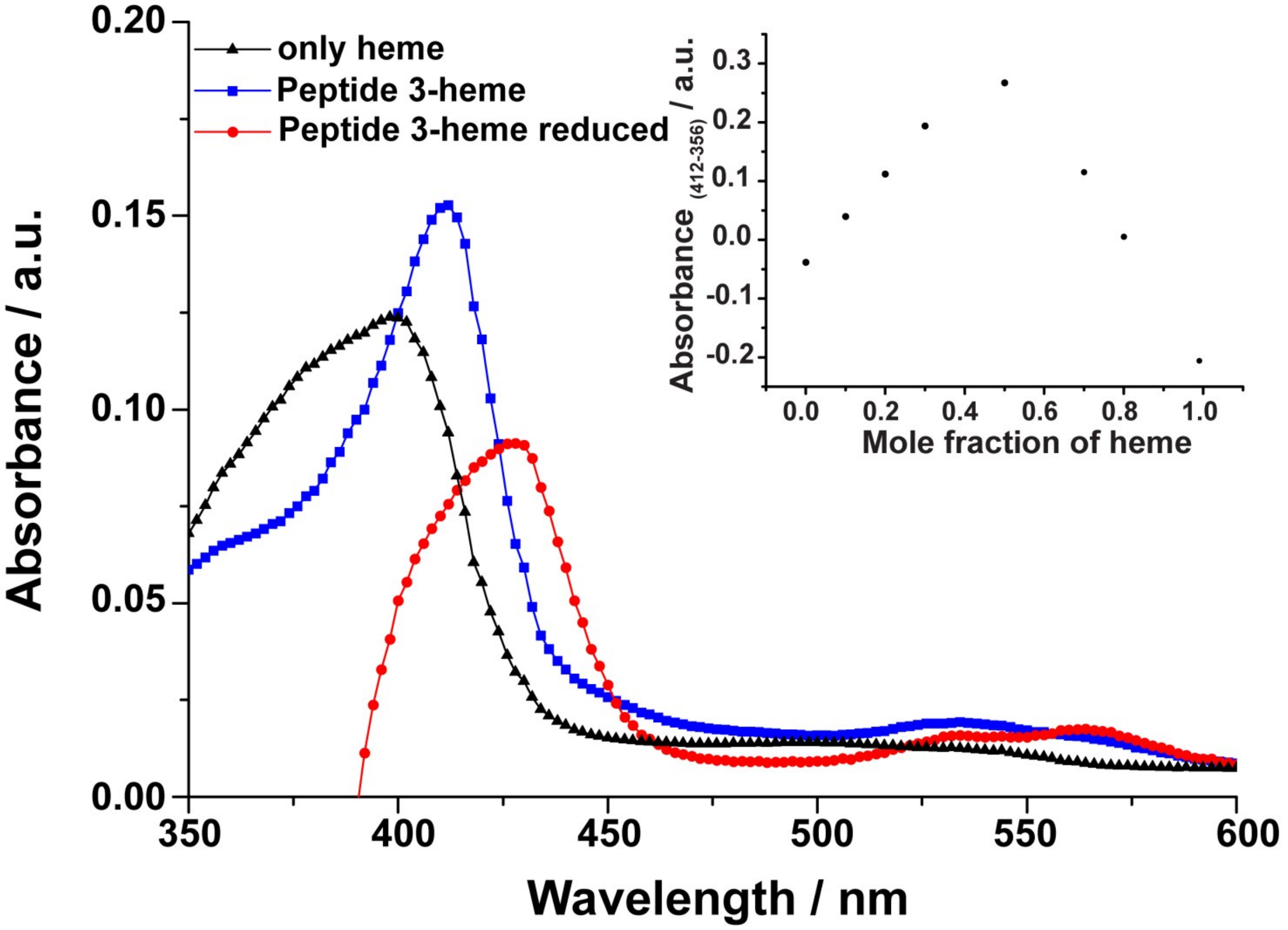


**Supplementary Figure 4:** Absorption spectra of heme alone (black), peptide 3-heme oxidized (blue) and peptide 3-heme reduced (red). Concentration of peptide and heme was 10 μM in 2 mM DPC, 50 mM sodium phosphate buffer, pH 7.2. Job's plot of peptide-3 (onset). The shift in Soret band of heme at 412 nm upon binding to peptide-3 and further shift to 428 nm after reduction by sodium dithionite and low intense peaks at 530 and 560 nm indicated bis-histidine coordination. The job plot shows peptide:heme stoichiometry 1:1.

**Figure S5**

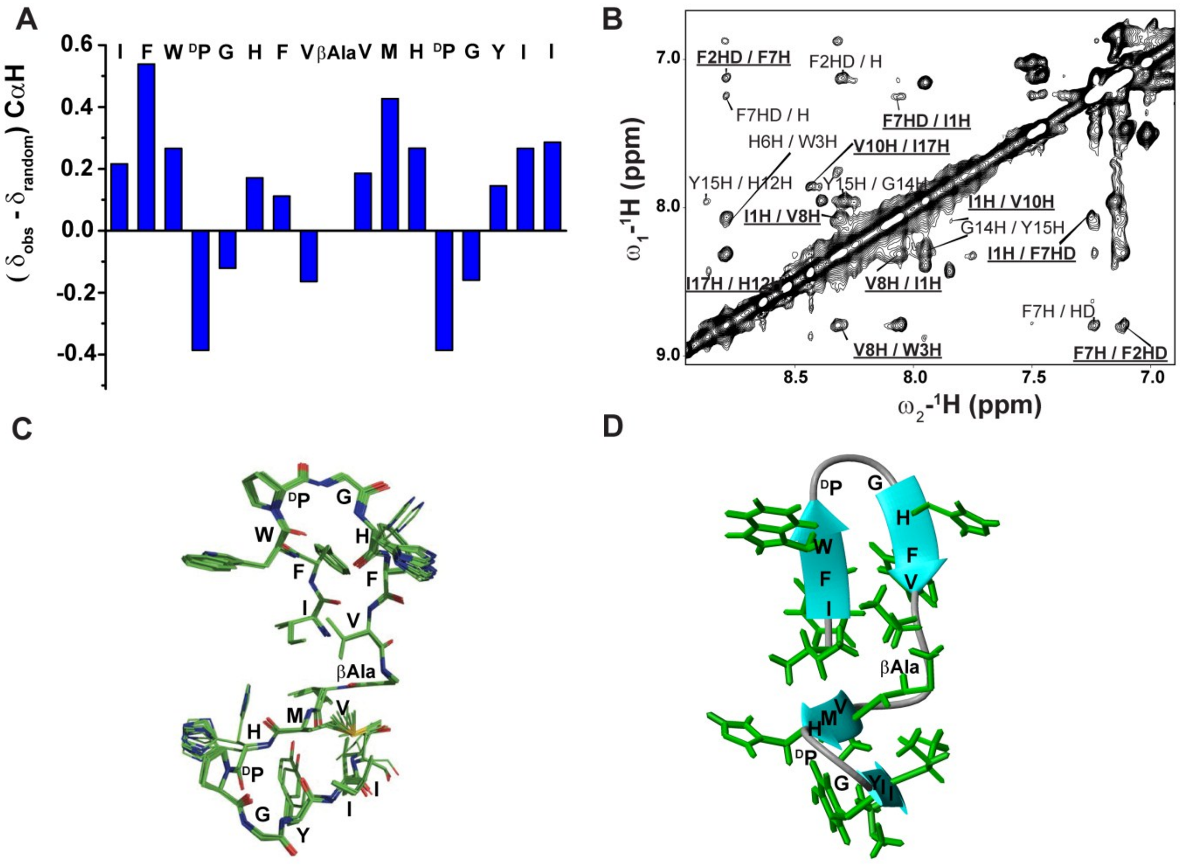


**Supplementary Figure 5:** (A) αH chemical shift deviation of peptide-3 (B) Section of two-dimensional $^1$H-$^1$H NOESY spectra showing NOE connectivity between amide protons. Long range NOEs are underlined and boldfaced. (C) Superimposed twenty low energy conformers of peptide-3. (D) A selected structure of peptide-3 showing side chain packing within β-sheets.

**Figure S6**

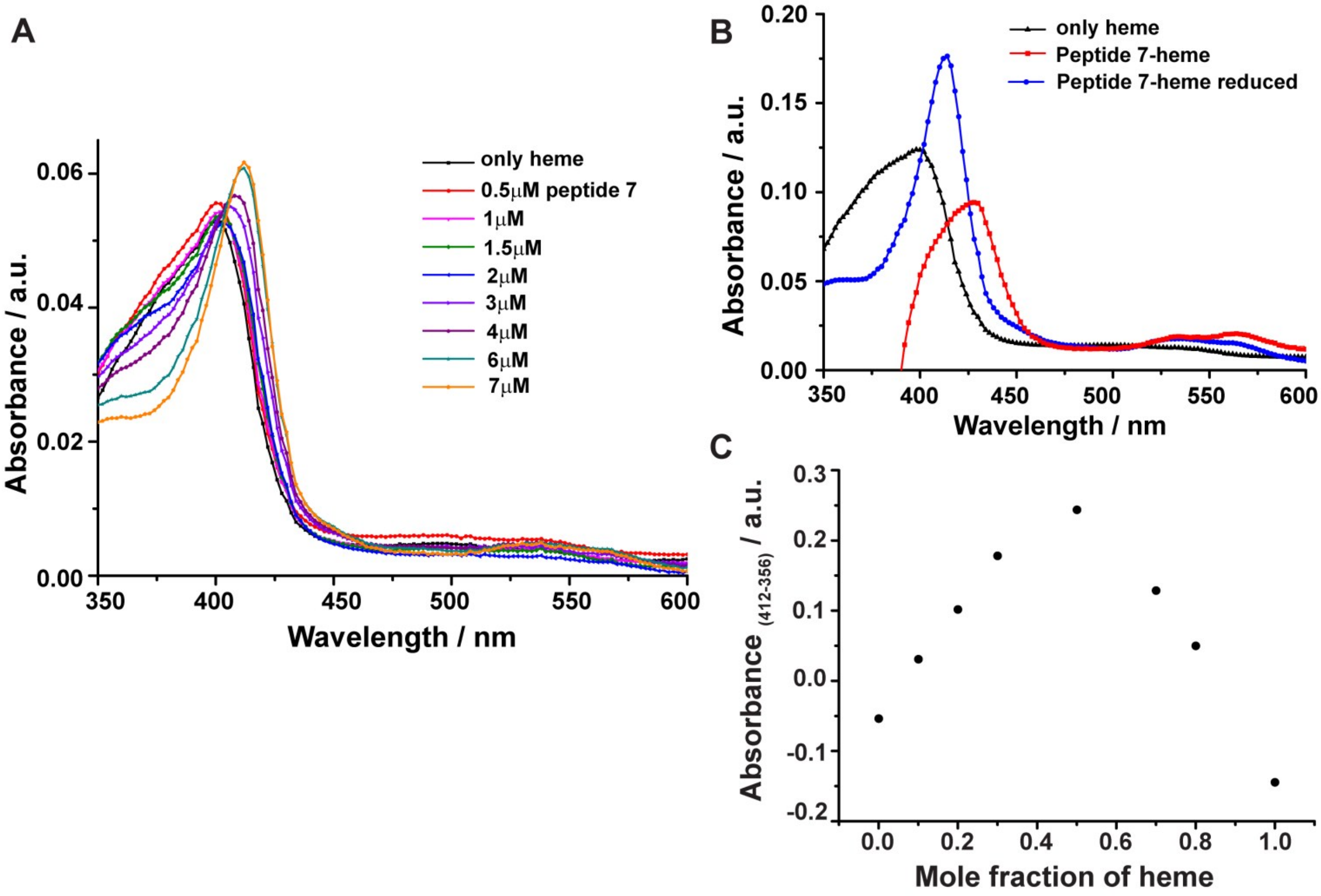


**Supplementary Figure 6:** (A) Absorption changes of heme (2 μM) on titrating peptide-7 (0-7 μM) in 50 mM sodium phosphate buffer, pH 7.2 containing 2 mM DPC. (B) Absorption spectra of heme alone (black), peptide7-heme oxidized (blue) and peptide7-heme reduced (red). Concentration of peptide and heme was 10 μM in 2 mM DPC, 50 mM sodium phosphate buffer, pH 7.2 (C) Jobs plot of peptide-7. The shift in Soret band of heme at 412 nm upon binding to peptide-3 and further shift to 428 nm after reduction by sodium dithionite and low intense peaks at 530 and 560 nm indicated bis-histidine coordination. The job plot shows peptide:heme stoichiometry 1:1.

Figure S7

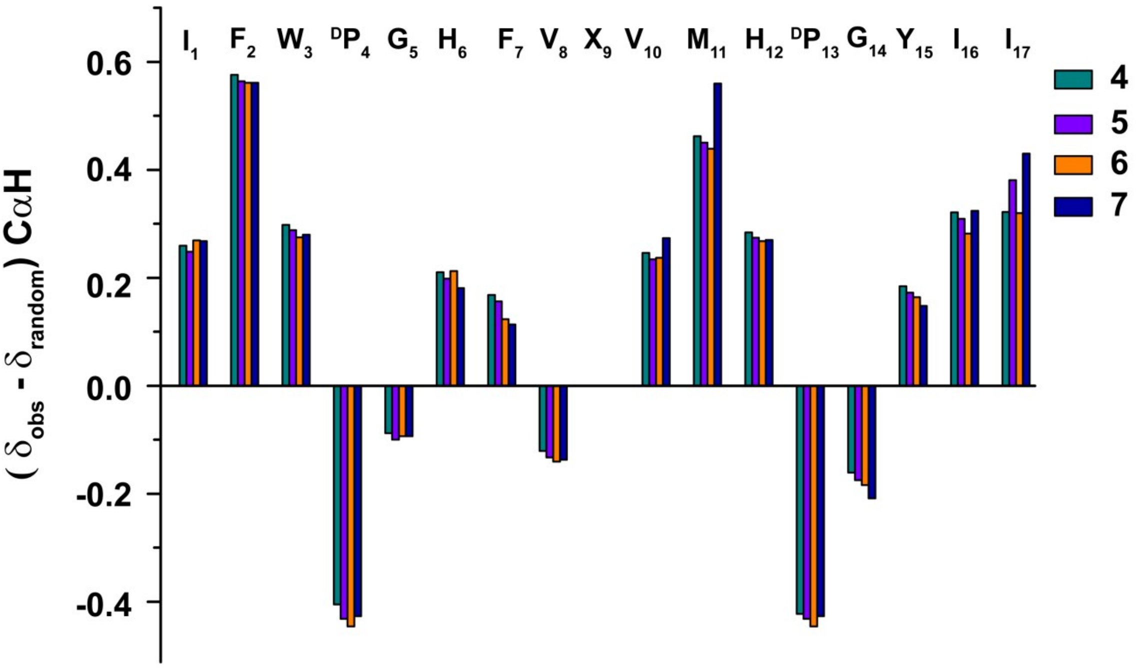


**Supplementary Figure 7:** Secondary chemical shift of αH peptide 4 to 7.

\

**Figure S8**

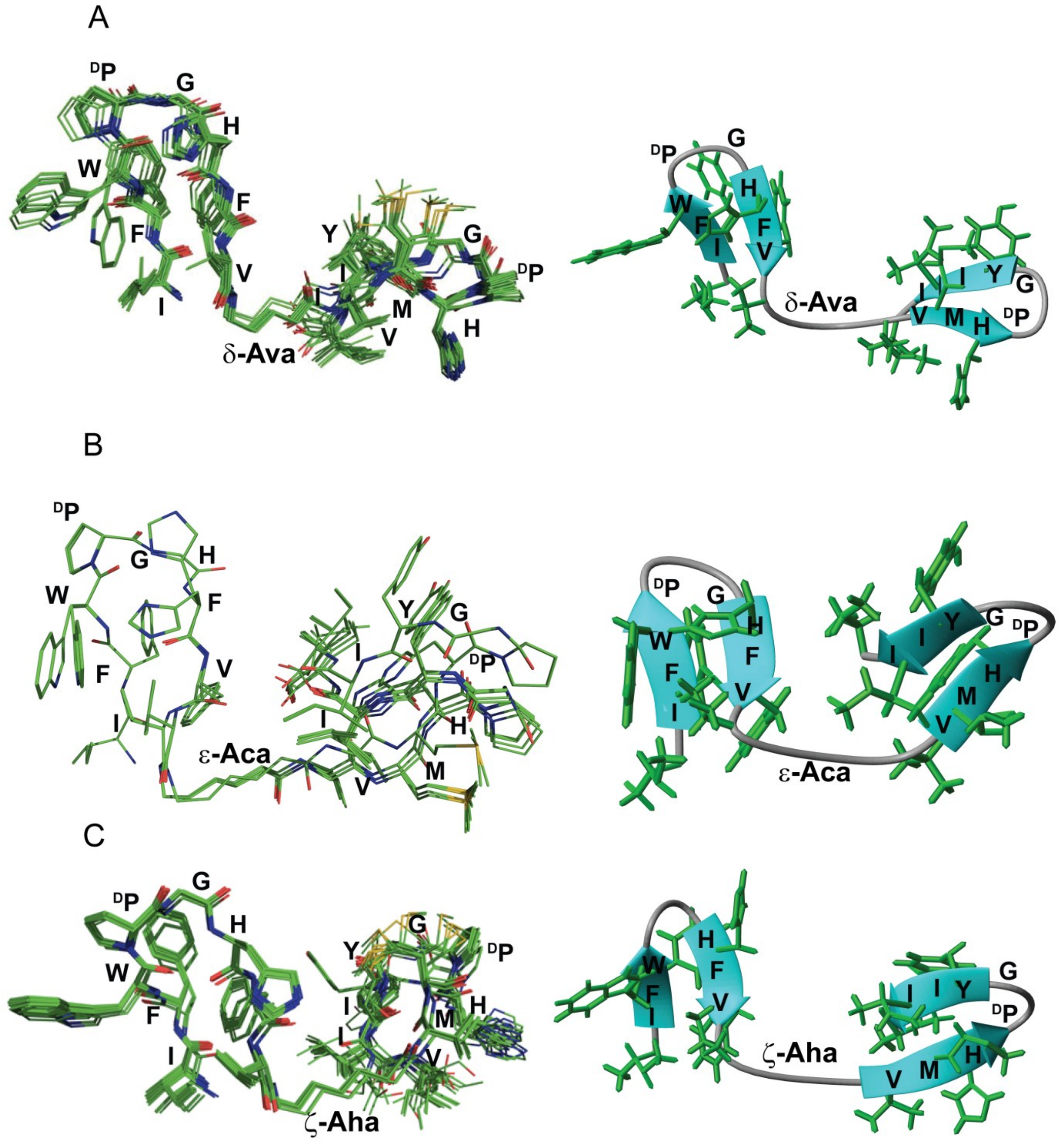


**Supplementary Figure 8:** Superimposed twenty low energy structures and one selected structure of each peptide showing side chain packing within β-sheets. (A) Peptide-4, (B) Peptide-5 and (C) Peptide-6

**Figure S9**

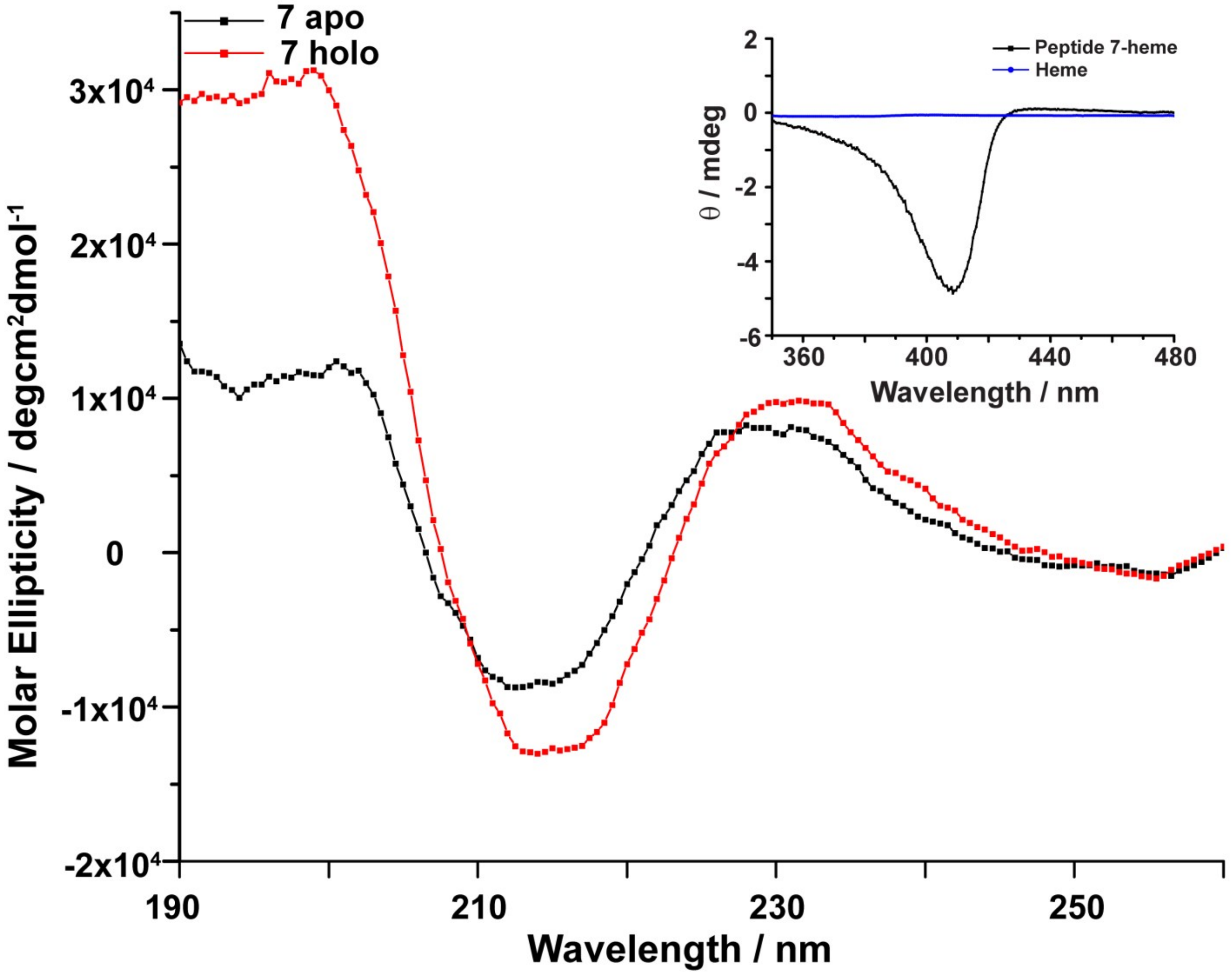


**Supplementary Figure 9:** Far-UV CD spectra of peptide-7 alone (black) and heme-bound (red). Concentration of peptide and heme was 100 μM in 2 mM DPC, 50 mM sodium phosphate buffer, pH 7.2. CD studies were performed using a 0.01cm path length cuvette with a 1nm bandwidth and a step size of 0.5 nm at 0.5 seconds per data set. Near-UV CD spectra of peptide-7-heme and heme alone (in blue) (onset) were acquired in a 1 cm path length cuvette to monitor the changes in heme environment. 8 μM of peptide along with 8 μM heme dissolved in 2 mM DPC, sodium phosphate buffer pH 7.2 was used for the experiment.

**Figure S10**

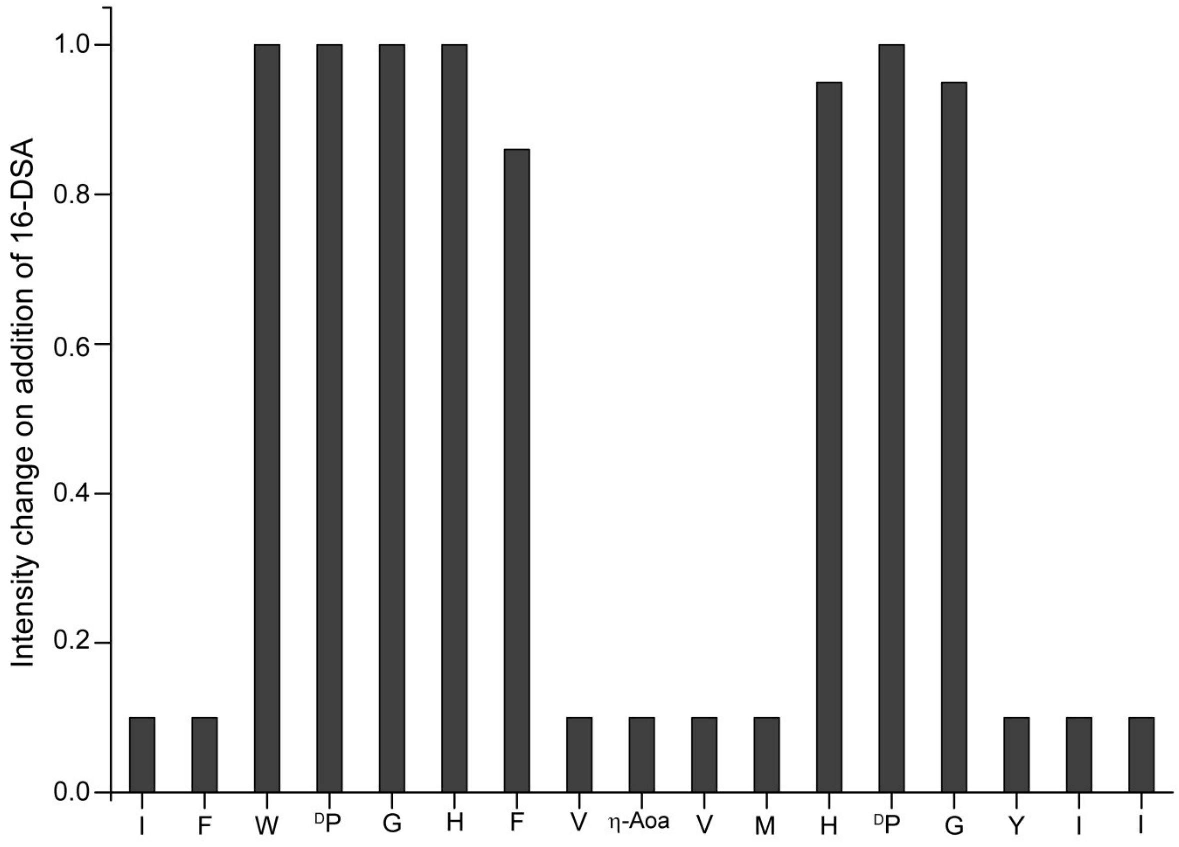


**Supplementary Figure 10:** The bar diagram showing ratio of intensity of CαH/NH cross-peaks obtained from two-dimensional TOCSY spectra of peptide-7 before addition of 16-DSA and in the presence of 16-DSA (2 mM). Residues experiencing perturbation by paramagnetic 16-DSA showed intensity ratio <1.

**Figure S11**

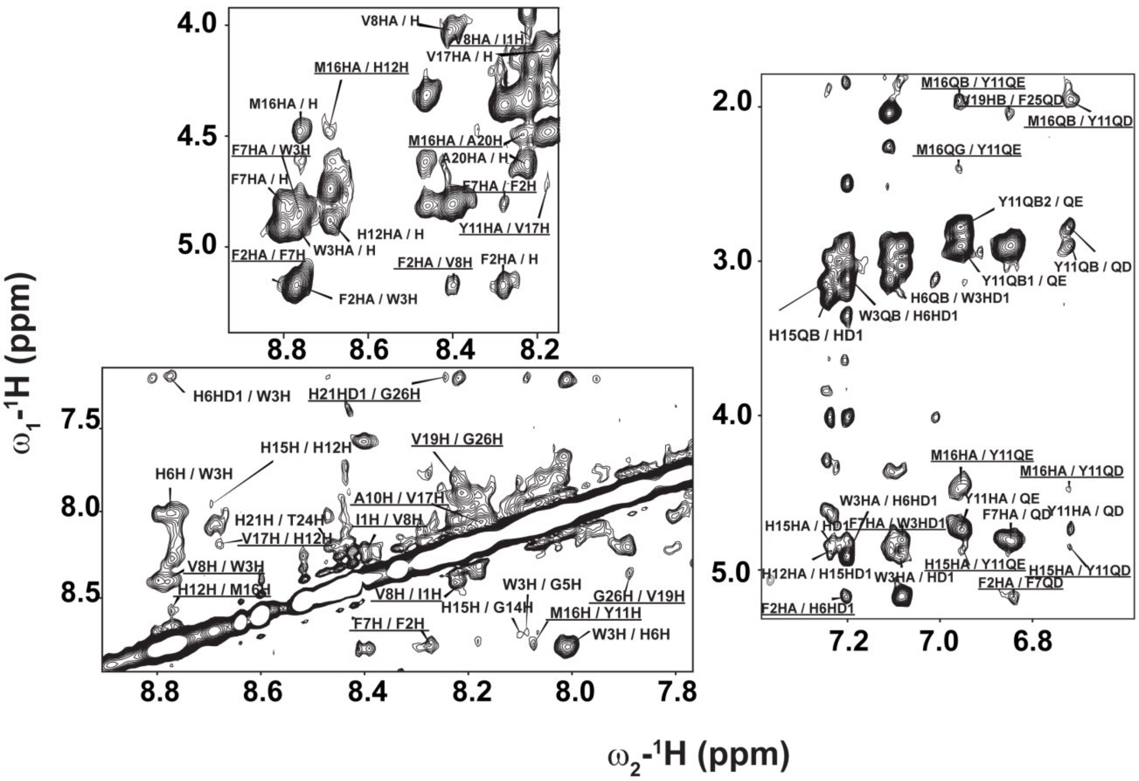


**Supplementary Figure 11:** Sections of two-dimensional $^1$H-$^1$H NOESY spectra of peptide-8 showing NOE connectivity in the $\alpha$H-NH region (top left), NH-NH region (bottom left) and aromatic region (right). Long range NOEs are underlined and boldfaced.

**Figure S12**

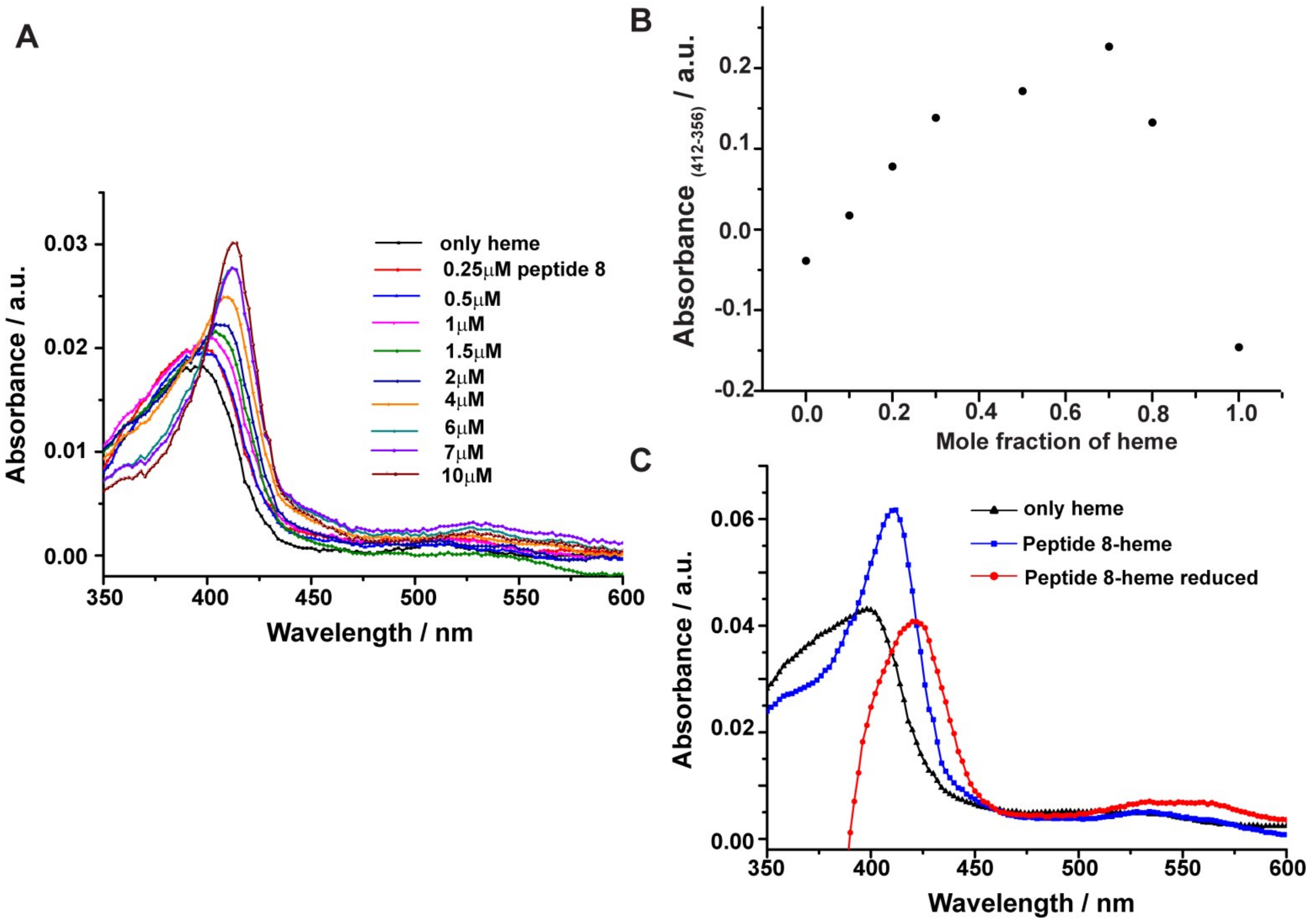


**Supplementary Figure 12:** (A) Absorption changes of heme (2 μM) on titrating peptide-8 (0-10 μM) in 50 mM sodium phosphate buffer, pH 7.2 containing 2 mM DPC. (B) Jobs plot of peptide-8. (C) Absorption spectra of heme alone (black), peptide 8-heme oxidized (blue) and peptide 8-heme reduced (red) Concentration of peptide and heme was 10 μM and 2 μM respectively in 2 mM DPC, 50 mM sodium phosphate buffer, pH 7.2. The shift in Soret band of heme at 412 nm upon binding to peptide-8 and further shift to 428 nm after reduction by sodium dithionite and low intense peaks at 530 and 560 nm indicated bis-histidine coordination. The job plot shows peptide:heme stoichiometry 1:2.

**Supplementary Table 1: Summary of structural statistics of the designed peptides**

| | 1 | 2 | 3 | 4 | 5 | 6 | 7 | 8 |
|---|---|---|---|---|---|---|---|---|
| **Distance constraints:** | | | | | | | | |
| Sequential [│i-j│ = 1] | 46 | 38 | 49 | 61 | 65 | 62 | 58 | 84 |
| Medium range [1<│i-j│< 4] | 12 | 6 | 12 | 13 | 19 | 10 | 16 | 26 |
| Long range [│i-j│ ≥ 4] | 14 | 19 | 20 | 28 | 21 | 25 | 32 | 38 |
| PRE driven distance restraints | | | | | | | 18 | |
| Total | 156 | 131 | 159 | 200 | 183 | 185 | 206 | 278 |
| **Dihedral-angle constraints** | 22 | 22 | 22 | 22 | 22 | 22 | 22 | 36 |
| **Deviation from mean structure** (Å) | | | | | | | | |
| All backbone atoms | 0.30 | 0.37 | 0.20 | 0.45 | 0.35 | 0.44 | 0.14 | 1.32 |
| All heavy atoms | 0.57 | 0.65 | 0.42 | 0.84 | 0.81 | 0.93 | 0.50 | 1.49 |
| **Ramachandran plot for the mean structure (%residues)** | | | | | | | | |
| Most favoured region | 92.3 | 75.0 | 92.3 | 92.3 | 92.3 | 92.3 | 92.3 | 72.6 |
| Additionally allowed region | 7.7 | 25.0 | 7.7 | 7.7 | 7.7 | 7.7 | 7.7 | 27.3 |
| Generously allowed region | 0 | 0 | 0 | 0 | 0 | 0 | 0 | 0 |
| Disallowed region | 0 | 0 | 0 | 0 | 0 | 0 | 0 | 0 |
